# Assessment of otolith function using vestibular evoked myogenic potential in women during pregnancy

**DOI:** 10.1016/j.bjorl.2020.08.003

**Published:** 2020-09-28

**Authors:** G. Bhavana, Kaushlendra Kumar, E. Anupriya

**Affiliations:** Manipal Academy of Higher Education, Manipal, Kasturba Medical College, Mangalore, Department of Audiology and Speech-Language Pathology, Karnataka, India

**Keywords:** cVEMP, oVEMP, Pregnancy, Vestibular, Otolith structure

## Abstract

**Introduction:**

More than 50% of pregnant women experience dizziness frequently in the first two gestational trimesters. During pregnancy, the changes in the metabolism of hormones are responsible for the ovarian cycle resulting in either peripheral or central vestibular alterations. The need for the study is to focus on the effect of changes during pregnancy on the vestibular evoked myogenic potential, an electrophysiological measure that investigates functions of the otolith structures.

**Objectives:**

The aim is to investigate the vestibular evoked myogenic potential responses during the first trimester of pregnancy.

**Methods:**

A total of 17 pregnant women and 17 non-pregnant women with age matched took part in this study. The cervical vestibular evoked myogenic potential were recorded from the ipsilateral sternocleridomastoid muscle and the ocular vestibular evoked myogenic potential were recorded from contralateral extraocular muscle in both groups.

**Results:**

Peak to peak amplitude of cervical vestibular evoked myogenic potential and ocular vestibular evoked myogenic potential was found to be significantly reduced in the responses obtained from first-trimester pregnant women when compared to that of non-pregnant women.

**Conclusions:**

Vestibular evoked myogenic potential tests exhibits a clinically significant reduced peak to peak amplitude in the first trimester of pregnancy, which indicates dysfunction in the otolith reflex pathway.

## Introduction

Pregnancy induces various physiological changes in almost all organ systems. Crucial changes exhibited during pregnancy include intracellular and extracellular fluid changes, osmolality changes, and alterations in the immune system. These changes in pregnancy may have impacts on cochlear microcirculation and fluid balance in the cochlea, which may alter hearing sensitivity.^1^, ^2^ Changes in the metabolism of steroid hormones (estrogen and progesterone), responsible for the ovarian cycle can also result in either peripheral or central vestibular alterations, which may occur during the normal menstrual cycle, gestation, menopause and the pre-menstrual periods.^3^ The symptoms are often associated with the action of estrogen and progesterone on the cochlea, posterior labyrinth, and central auditory pathways with hearing and balance alterations, which are clinically referred to as vertigo, instability, tinnitus, ear fullness, or hyperacusis.^3^, ^4^ More than 50% of pregnant women experience dizziness which occurs more frequently in the first two gestational trimesters. The hormonal alteration during pregnancy leads to possible vestibular alterations resulting in dizziness associated with nausea during the first gestational trimester, and this complaint diminishes in the following trimesters because of labyrinthine habituation.^3^ And there is very limited evidence that explains the effects of hormonal changes during pregnancy on the otolithic organs of the vestibular system.

To investigates the functions of the otolithic structures, inferior,^5^, ^6^ and the superior branch of the vestibular nerve^7^ an electrophysiological measure vestibular evoked myogenic potential (VEMP) is used. The VEMPs responses are obtained from the otolithic organs, the utricle, and the saccule, through the vestibulo-ocular and vestibulo-collic reflexes respectively.^8^ The vestibulo-collic reflex responses are obtained from cervical VEMP testing (cVEMP) having its origin in the vestibular saccule further carried by the inferior vestibular nerve and finally synapsing at the sternocleidomastoid muscle.^9^, ^10^ The vestibulo-ocular reflex pathway responses are acquired from ocular VEMP testing (oVEMP) which is presumed to have its origin from the utricle.^11^ The pathway is thus thought to travel from the utricle, passing through the superior vestibular nerve, vestibular nucleus, then crossing over to the contralateral oculomotor nuclei via the medial longitudinal fasciculus and finally innervating the extraocular muscles.^8^ As there was prevailing evidence of physiological and metabolic changes in pregnant women within the vestibular function, there is a need to provide an insight into the effects of changes during pregnancy on the VEMP recordings. Hence, the present study aims to investigate the VEMPs responses during the first trimester of pregnancy. The first objective of the study is to compare cVEMP responses among pregnant women and non-pregnant women, and the second objective is to compare oVEMP responses between the two same groups.

## Methods

A cross-sectional study was initiated with the ethical approval from the Institutional Human Research Ethical Committee (IEC KMC MLR 11-15/268), and all participants were enrolled following informed consent. A total number of 34 participants were selected using convenient sampling with a mean age of 27.68 ± 4.48 years, among which 17 were the first-trimester pregnant women and 17 age-matched non-pregnant women. The criteria employed were the individuals in the first trimester of pregnancy with normal external, middle, and inner ear function and with no history of any disease condition (H.E.L.L.P, jaundice, measles, etc.) before or during their pregnancy. The participant should not have any history of neurological and systemic illness.

### Procedure

This study was carried out in two phases. Phase 1 was the pre-assessment for the selection of participants, and Phase 2 was the experimental task. Phase 1 consisted of pre-assessment tests which included detailed individual case history, otoscopic examination, pure tone audiometry, and immittance. Phase 2 consisted of the actual experiment involving cVEMP and oVEMP testing. All the tests were carried out in a sound-treated room.

#### Pre-assessment for selection of participants

Initially, a detailed case history was obtained from each participant, followed by an otoscopic examination to ascertain that the ear canal was free from wax. Subsequently, pure tone audiometry was performed. A calibrated diagnostic audiometer (GSI-61) was used to estimate both air conduction and bone conduction pure tone thresholds and UCL (uncomfortable level). Pure tone audiometry was done by using the modified Hughson-Westlake procedure.^12^ Across octave frequencies from 250 Hz to 8 kHz for air conduction, and from 250 Hz to 4 kHZ for bone conduction was used to obtain the auditory thresholds. The UCL was obtained before VEMP testing as the latter requires a stimulus presentation of 500 Hz tone burst at a high-intensity level (105 dBnHL). UCL was tracked using the ascending method by presenting a 500 Hz pure tone through TDH-39 headphones at high intensities at the suprathreshold level. GSI tympstar was used for immittance measurement. Immittance evaluation was performed using a 226 Hz probe tone to ensure that the participants had normal middle ear function. A type ‘A’ tympanogram with the presence of ipsilateral and contralateral acoustic stapedial reflexes at octave frequencies ranging from 500 Hz to 4 kHz was obtained for all participants. The above-mentioned procedures were performed to meet the subject inclusion criteria.

#### Experimental tasks

These studies consisted of cVEMP and oVEMP testing. VEMP testing was done on the participants who fulfilled the above-mentioned inclusion criteria. VEMP testing was done using the Intelligent Hearing System (IHS) (version 3.92) in an electrophysiological laboratory. Ear-Tone 3A insert earphones were used to deliver the stimuli. The participants were seated in an upright relaxed position. The participants were informed regarding the testing procedure of method of cleaning and placement of electrodes at the various location for the recording of cVEMP and oVEMP. The responses were recorded using gold-plated electrodes. Before placing the electrodes, the sites for electrode placements were cleaned using Nu-prep skin conduction paste, and electrodes were placed with the ten-20 gel to increase conductivity. Blackman window 500 Hz tone burst with 7 ms duration was used as a stimulus and presented monaurally through an insert earphone at a stimulus intensity level of 105 dBnHL. The waveform replicability was checked by taking two recordings. A total of 200 sweeps of rarefaction stimuli were presented at a repetition rate of 5.1/s. A time window of 60 ms (pre-stimulus −10 ms & post-stimulus 50 ms) was incorporated. The absolute electrode impedance was maintained so that the impedance at each electrode site was less than 5 kΩ and inter-electrode impedance within 3 kΩ for both cVEMP and oVEMP recording.

### Cervical vestibular evoked myogenic potential (cVEMP) testing

The electrode montage included the placement of the non-inverting electrode on the midpoint of the sternocleidomastoid muscle of the test ear, with the inverting electrode on the sternoclavicular junction and the ground electrode on the forehead. The electromyographic signals were amplified 5000 times and bandpass filtered between 30 and 1500 Hz. The participants were instructed to turn their heads to the contralateral side of the test ear to maintain constant tonic muscle activity of the ipsilateral SCM muscle between 50 µV to 150 µV throughout the recording. By providing visual feedback to the participant, the software ensured that sufficient muscle contraction was achieved throughout the testing. Whenever the tonic muscle contraction level fell below 50 µV or above 150 µV, the responses were rejected. The biphasic wave with a Positive (P1) and a subsequent Negative (N1) peak were recorded to determine the latency and peak to peak amplitude for bilateral responses.

### Ocular vestibular evoked myogenic potential (oVEMP) testing

The electrode configuration involved the placement of a non-inverting electrode beneath the eye, contralateral to the ear being tested. The inverting electrode was positioned 1–2 cm below the non-inverting electrode over the cheek and the ground electrode was placed on the forehead. oVEMP was recorded from the contraction of contralateral extraocular muscle. The EMG signals were amplified 50,000 times and bandpass filtered between 1 Hz–1500 Hz. The participants were instructed to look upward at a visual angle of 30–35° vertically above at a fixed target which was approximate >2-m distance from the eyes. The recording was initiated and the initial biphasic wave with a negative (n1) peak followed by a positive peak (p1) was used to determine the latency and peak to peak amplitude for bilateral responses.

### Statistical analysis

The cVEMP and oVEMP waveform obtained from pregnant women and non-pregnant women were analyzed for latency and amplitude measures. Latency measures included P1 and N1 latencies for cVEMP and n1 and p1 latencies for oVEMP. Peak to peak amplitude of P1–N1 and n1–p1 was analyzed for cVEMP and oVEMP responses, respectively. This obtained data was then tabulated and statistically analyzed using the statistical software SPSS version 16.0. Mann–Whitney *U* test was done to see the significant difference between the two groups.

## Result

### cVEMP

**Figure 1:**
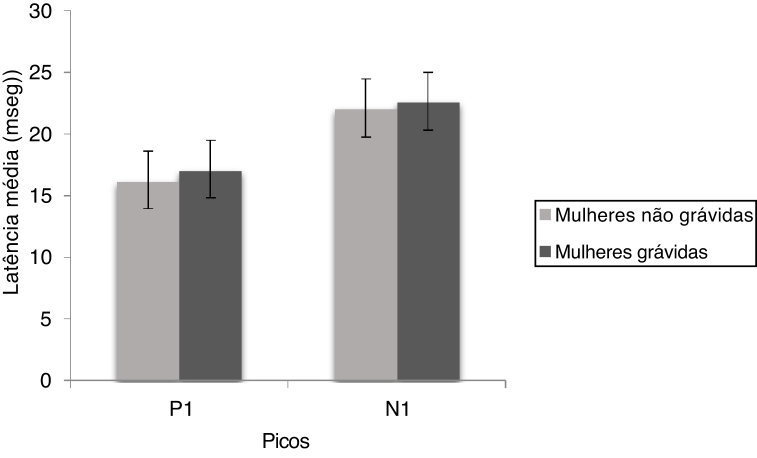
The mean and standard deviation of P1 and N1 latencies of cVEMP for non-pregnant and pregnant women.

The mean and standard deviation of P1 and N1 latencies of cVEMP are shown in Fig. 1. it is observed that the P1 and N1 latencies were similar for both the groups and the Mann Whitney test showed there was no significant difference for P1 (U = 449.00, *p* = 0.114) and N1 (U = 478.00, *p* = 0.220) latencies between non-pregnant and pregnant women.

**Figure 2:**
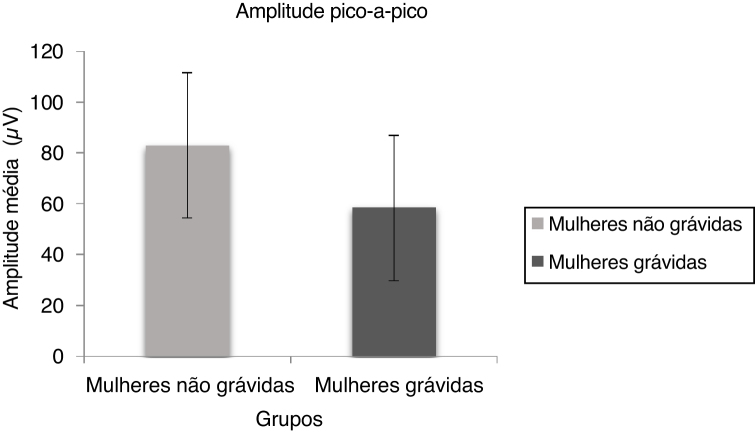
The mean and standard deviation of P1–N1 amplitude of cVEMP for non-pregnant and pregnant women.

Fig. 2 depicts the mean and standard deviation of the peak to peak amplitude for pregnant women and non-pregnant women. Mann Whitney test results indicate there was a significant difference in peak to peak amplitude (U = 345.00, *p* = 0.004) between non-pregnant and pregnant women.

### oVEMP

**Figure 3:**
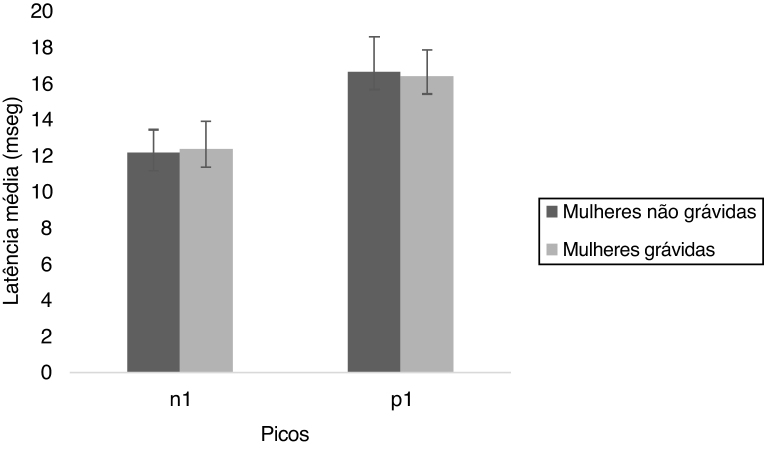
The mean and standard deviation of n1 and p1 latencies of oVEMP for non-pregnant and pregnant women.

From the Fig. 3, it is observed that the n1 and p1 latencies were similar for both the groups. The Mann Whitney test showed that there was no significant difference for n1 (U = 456.00, *p* = 0.258) and p1 (U = 525.50, *p* = 0.812) latencies between non-pregnant and pregnant women. The results indicate that the n1 and p1 latencies were similar for both the groups.

**Figure 4:**
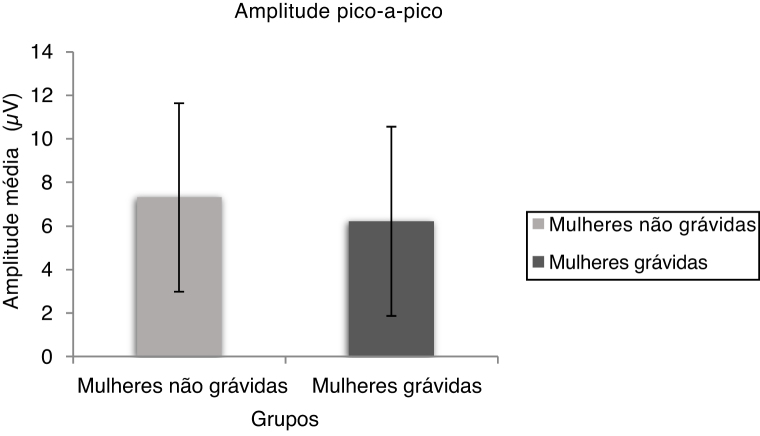
The mean and standard deviation of n1–p1 amplitude of oVEMP for non-pregnant and pregnant women.

The peak to peak amplitude means and standard deviation of oVEMP are shown in Fig. 4 for non-pregnant and pregnant women. The Mann Whitney test results indicate a significant difference in peak to peak amplitude (U = 355.00, *p* = 0.015) between non-pregnant and pregnant women.

## Discussion

This study revealed that the peak to peak amplitude of cVEMP and oVEMP responses was found to be significantly reduced in the first trimester pregnant women when compared to that of non-pregnant women. The reviews on test-retest reproducibility studies showed that peak to peak amplitude of VEMP responses seems to have good significance.^13^, ^14^ In the present study, the reduction in peak to peak amplitude of VEMPs in pregnant women may be due to alterations in the otolithic organ, and such alterations in the vestibular system can be attributed to the hormonal milieu changes that occur during pregnancy. Supporting the findings of the present study, there is literature showing that the neurotransmitters released during pregnancy can alter the biochemical control of the inner ear, which possibly triggers an increase in neurotological symptoms.^3^ Such a fact can be responsible for the frequent complaint of dizziness during pregnancy. During pregnancy, the levels of both ovarian hormones are higher than usual, and there can be signs of other complex physiological changes also.^15^ The receptors present in the spiral ganglion and the hair cells (OHC and IHC’s) theorize that estrogen may affect the auditory transmission, whereas the fluid-electrolyte balance in the cochlea is affected by the receptors in the stria vascularis.^16^ Progesterone is an ovarian hormone emanated during the luteal phase of the ovarian cycle by the corpus luteum, which acts as a neurosteroid and forms a progenitor to other steroid hormones.^17^ Progesterone along with its metabolites may also affect the auditory system when it interacts with the steroid-binding sites as GABA-A antagonists on the GABA-A receptors.^18^ Thus, in general, progesterone is mainly found to have an inhibitory action on the CNS, counteracting the estrogen’s excitatory action, leading to a balance in the auditory system.

Schmidt et al., had reported that 52.44% of women had dizziness during pregnancy among which the highest (63.64%) occurrence was observed during the first trimester followed by second (60.61%) trimester. During the third trimester, dizziness was reported to be present in only 33.33% in pregnant women. The symptom of vestibular alteration can be due to the hormonal changes and the reduction in the symptom in the following trimesters happens due to labyrinthine habituation.^3^ Another study reports that vestibular alteration normalizes throughout the gestational period which may be attributed to the labyrinthine habituation.^19^ Nausea and vomiting during pregnancy occurs in 85% of pregnant women, with varying degrees of severity having been reported.^20^ On the other hand, another study suggested that the etiology is unknown but most likely to be multi-factorial, such as hormonal alternation, thyroid disorders, vitamin deficiency, etc. Effects of pregnancy on Meniere’s disease report that vertigo attack increased up to ten times per month during early pregnancy because of significantly reduced serum osmolality which normalizes in the following trimesters.^21^

In the present study, the reduction in VEMPs amplitude suggestive of peripheral labyrinthine dysfunction. The literature reveals that, during pregnancy, there are hormonal abnormalities or changes which act as a predisposing factor for dizziness. During the menstrual cycle, gestation, and menopause there are hormonal alterations that cause various homeostatic, metabolic effects. Numerous theories have been proposed concerning estrogen effects. The estrogen receptors have been identified, mainly in the spiral ganglion and stria vascularis, which are important in hearing transmission and inner ear homeostasis.^22^ There is evidence stating that estrogen affects endolymph ionic and anionic homeostasis by regulating ion and anion channels.^23^, ^24^ Alteration in estrogen causes otoconial degeneration and detachment leading to positional vertigo.^16^

## Conclusion

The results of this investigation have shown reduced peak to peak amplitude during the first trimester of pregnancy. The reduction in amplitude has been observed in of cVEMP and oVEMP. The reduction in amplitude indicates dysfunction in the otolith reflex pathway. As pregnant women usually have vestibular complaints, VEMP can be used as a clinical tool for investigation and further monitoring can be done if necessary.

## Conflicts of interest

The authors declare no conflicts of interest.
